# The Necessity of Dissection of No. 14 Lymph Nodes to Patients With Pancreatic Ductal Adenocarcinoma Based on the Embryonic Development of the Head of the Pancreas

**DOI:** 10.3389/fonc.2020.01343

**Published:** 2020-08-11

**Authors:** Lihan Qian, Junjie Xie, Zhiwei Xu, Xiaxing Deng, Hao Chen, Chenghong Peng, Hongwei Li, Weimin Chai, Jing Xie, Weishen Wang, Baiyong Shen

**Affiliations:** ^1^Departement of General Surgery, Pancreatic Disease Center, Ruijin Hospital, School of Medicine, Shanghai Jiao Tong University, Shanghai, China; ^2^Research Institute of Pancreatic Disease, School of Medicine, Shanghai Jiao Tong University, Shanghai, China; ^3^State Key Laboratory of Oncogenes and Related Genes, Shanghai Jiao Tong University, Shanghai, China; ^4^Department of Radiology, Ruijin Hospital, School of Medicine, Shanghai Jiao Tong University, Shanghai, China; ^5^Department of Pathology, Ruijin Hospital, School of Medicine, Shanghai Jiao Tong University, Shanghai, China

**Keywords:** pancreas head cancer, pancreatic ductal adenocarcinoma (PDAC), pancreatic embryology, lymph node dissection (LN dissection), lymph nodes around superior mesenteric artery (SMA)

## Abstract

**Objectives:** Pancreaticoduodenectomy (PD) followed by lymphadenectomy is performed for patients with pancreatic ductal adenocarcinoma (PDAC) located in the head of the pancreas. Because the head of the pancreas could be divided into dorsal or ventral primordium in relation to embryonic development, the metastasis of lymph node (LN) may differ. In this retrospective study, we evaluated the impact of extended or standard LN dissection for PDAC located in ventral or dorsal primordia of the pancreatic head.

**Methods:** From February 2016 to November 2018, 178 patients who underwent PD for PDAC were enrolled at the Pancreatic Disease Center, Ruijin Hospital, School of Medicine, Shanghai Jiao Tong University. According to the tumor location and the range of LN dissection, all patients were divided into three groups: ventral primordium with extended lymphadenectomy (VE group), ventral primordium with standard lymphadenectomy (VS group), and dorsal primordium with extended lymphadenectomy (DE group). Clinical and pathological features were retrospectively analyzed as were the long-term survival outcomes.

**Results:** More patients in the VE group were detected with metastasis in the lymph nodes around the superior mesenteric artery (LN14) than those in the DE group (LN along the right side of the superior mesenteric artery, LN14ab): 22.9 vs. 5.9%, *p* = 0.005; (LN along the left side of the superior mesenteric artery, LN14cd): 10.0 vs. 0.0%, *p* = 0.022. LN14 was involved in more patients in the VE group than in the VS group (22.9 vs. 5.0%, *p* = 0.015). For IIb-stage patients in the VE group, the overall survival time (18.3 vs. 9.3 months, *p* < 0.001) and disease-free survival time (12.2 vs. 5.1 months, *p* = 0.045) were longer in those with LN14cd (–) than those with LN14cd (+).

**Conclusion:** This study suggested that patients with PDAC located in the ventral head of the pancreas had higher risk of LN14 involvement compared with those at dorsal. Thus, a thorough dissection of LN14 in PDAC located in the ventral head of the pancreas is recommended to optimize the regional extended lymphadenectomy.

## Introduction

Pancreatic cancer is a highly malignant digestive cancer with a median 5-years survival rate range from 2 to 9% (1, 2). Pancreatic ductal adenocarcinoma (PDAC) is the most frequent type, representing 60%−70% of pancreatic head neoplasms (3). Surgery is the main curative treatment for PDAC. Pancreaticoduodenectomy (PD) associated with standard or extended lymphadenectomy is recommended for patients with PDAC located in the head of the pancreas. Lymphadenectomy is an indispensable part in the curative pancreatic surgery, and lymph node (LN) metastasis has been recognized as one of the strongest prognostic factors. It has been shown that high-grade LN stage according to the American Joint Commission on Cancer (AJCC), 8th edition, predicts poor survival outcomes (4). The appropriate extent of lymphadenectomy to obtain a better prognosis has been the focus of clinical research.

The extent of standard lymphadenectomy of pancreatic head carcinomas includes the LNs station involved in two main routes of LN metastases: from the head of the pancreas to the common hepatic artery (CHA) then celiac axis and from the head of the pancreas to the superior mesenteric artery (SMA) (5). Furthermore, a previous study has demonstrated that PDAC located in the dorsal head of the pancreas are more likely to spread through the LNs of CHA and the hepatic duodenal ligament, and those located in the ventral head of the pancreas tended to spread through the LNs of SMA in relation to embryonic development (6).

The clarification of the profile of LNs, which are prone to metastasize according to the location of pancreatic head cancer, could help to optimize the surgical strategies and the prognosis of patients as well. Therefore, we conducted a retrospective study to investigate the lymphadenectomy strategies for PDAC in the head of the pancreas and their prognostic factors.

## Methods and Materials

### Patients and Data Source

**Figure 1:**
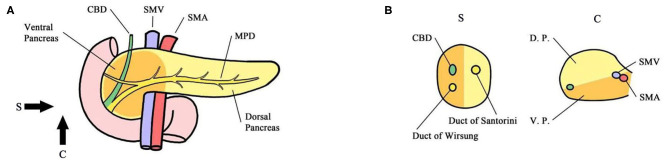
**(A)** Sagittal view. **(B)** Coronary view. The pancreas is codeveloped from the ventral and dorsal primordium, which mainly constitute the body and tail of the pancreas and anterior partial head of the pancreas (yellow). The ventral primordium develops into the posterior part of the head of the pancreas that surrounds SMA/SMV. The head of the pancreas was divided into the ventral and dorsal pancreatic head by the line that links the portal vein (PV)/superior mesenteric vein (SMV) and anterior edge of the intrapancreatic bile duct. The main pancreatic duct of the common bile duct was located in the ventral pancreatic head, and the accessory pancreatic duct was located in the dorsal pancreatic head. *CBD* Common Bile Duct *MPD* Main Pancreatic Duct *SMA* Superior Mesenteric Artery *SMV* Superior Mesenteric Vein *DP* Dorsal Primordium *VP* Ventral Primordium.

**Figure 2:**
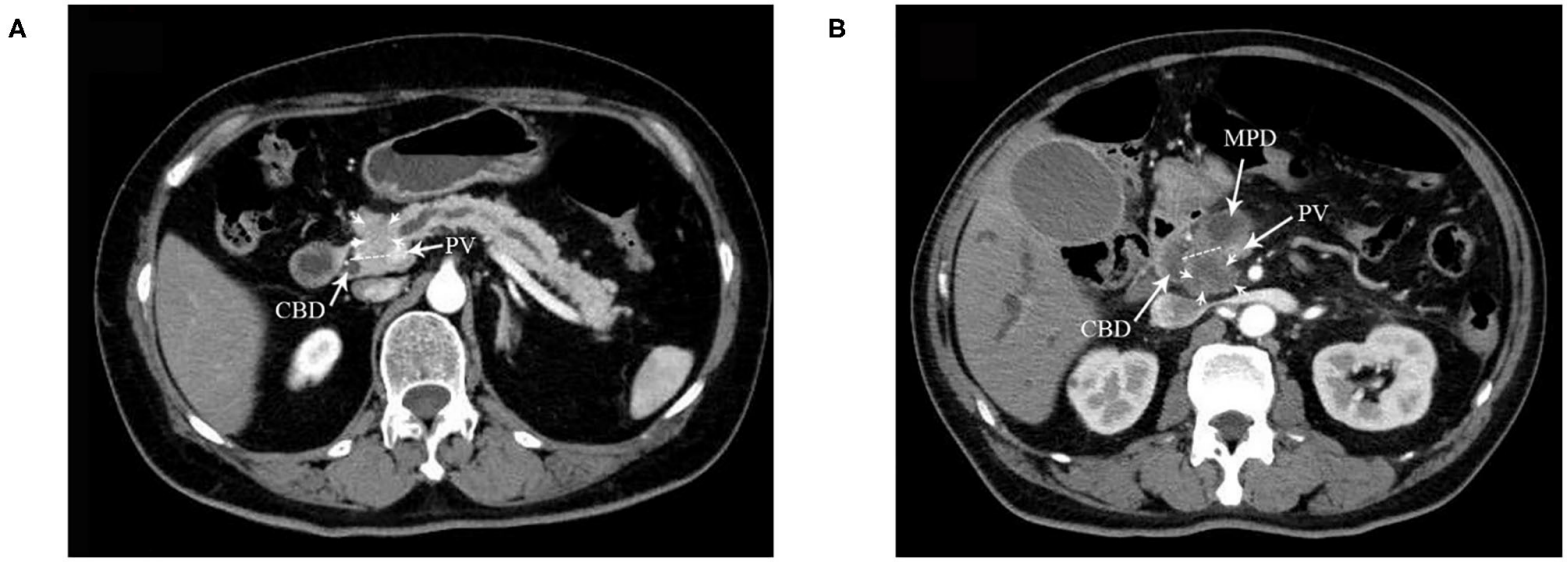
A dotted line on the CT image indicates the boundary between the ventral and dorsal head of the pancreas in **(A,B)**. **(A)** DE group: tumor located in the dorsal head of the pancreas. CBD, common bile duct. PV portal vein. Arrows indicate the tumor. **(B)** VE and VS groups: tumor located in the ventral head of the pancreas. MPD, mean pancreatic duct. Arrows indicate the tumor.

**Figure 3:**
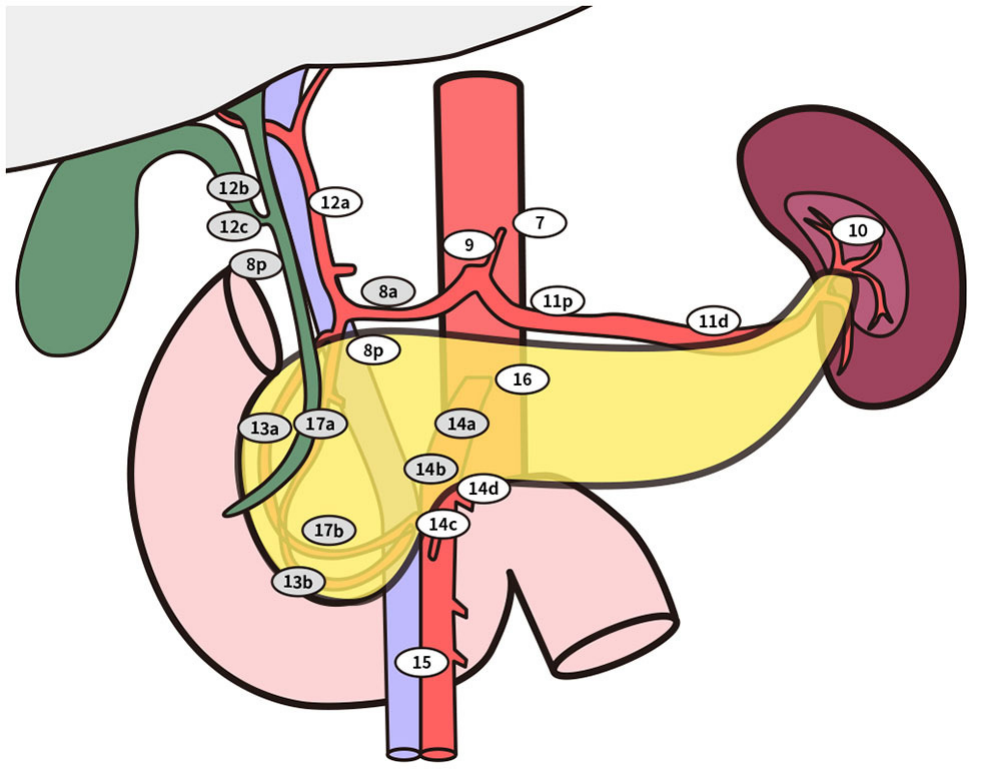
**Standard lymphadenectomy**. No. 5 Supra pyloric lymph nodes; No. 6 infra pyloric lymph nodes; No. 8a lymph nodes in the anterosuperior group along the common hepatic artery No. 12b lymph nodes along the bile duct; No. 12c (located next to 12b), lymph nodes around the cystic duct; No. 13a lymph nodes on the posterior aspect of the superior portion of the head of the pancreas; No. 13b lymph nodes on the posterior aspect of the inferior portion of the head of the pancreas; No. 14a-b lymph nodes along right side of superior mesenteric artery No. 17a lymph nodes on the anterior surface of the superior portion of the head of the pancreas; No. 17b lymph nodes on the anterior surface of the inferior portion of the head of the pancreas. **Extended lymphadenectomy**. No. 8p lymph nodes in the posterior group along the common hepatic artery; No. 12a lymph nodes along the hepatic artery; No. 12p lymph nodes along the portal vein; No. 14c-d lymph nodes along the left side of superior mesenteric artery; No. 16 lymph nodes around the abdominal aorta besides standard range of lymph node dissection.

Five hundred twenty-eight patients who were included in a formed randomized controlled trial (NCT02787187), which was designed to verify the survival benefit of extended lymphadenectomy at the Pancreatic Disease Center, Ruijin Hospital, School of Medicine, Shanghai Jiao Tong University from February 2016 to November 2018, were screened as follows. Inclusion criteria: (1) the carcinoma could be divided into either ventral or dorsal pancreatic head by a line that links the portal vein (PV)/superior mesenteric vein (SMV) and anterior edge of the intrapancreatic bile duct (Figures 1, 2) (6). (2) Patients with a tumor located in the ventral pancreas had performed standard or extended lymphadenectomy and those with a tumor located in the dorsal pancreas underwent extended lymphadenectomy (Figure 3). (3) All patients were pathologically diagnosed with PDAC. (4) The neoplasms were resectable conforming to the consensus proposed by the National Comprehensive Cancer Network without neoadjuvant chemotherapy (7). Exclusion criteria were (1) the intraoperative surgical margin was positive. (2) Distant metastases were confirmed intraoperatively. (3) Postoperative pathology confirmed the metastasis in para-aortic LN (LN16).

**Figure 4:**
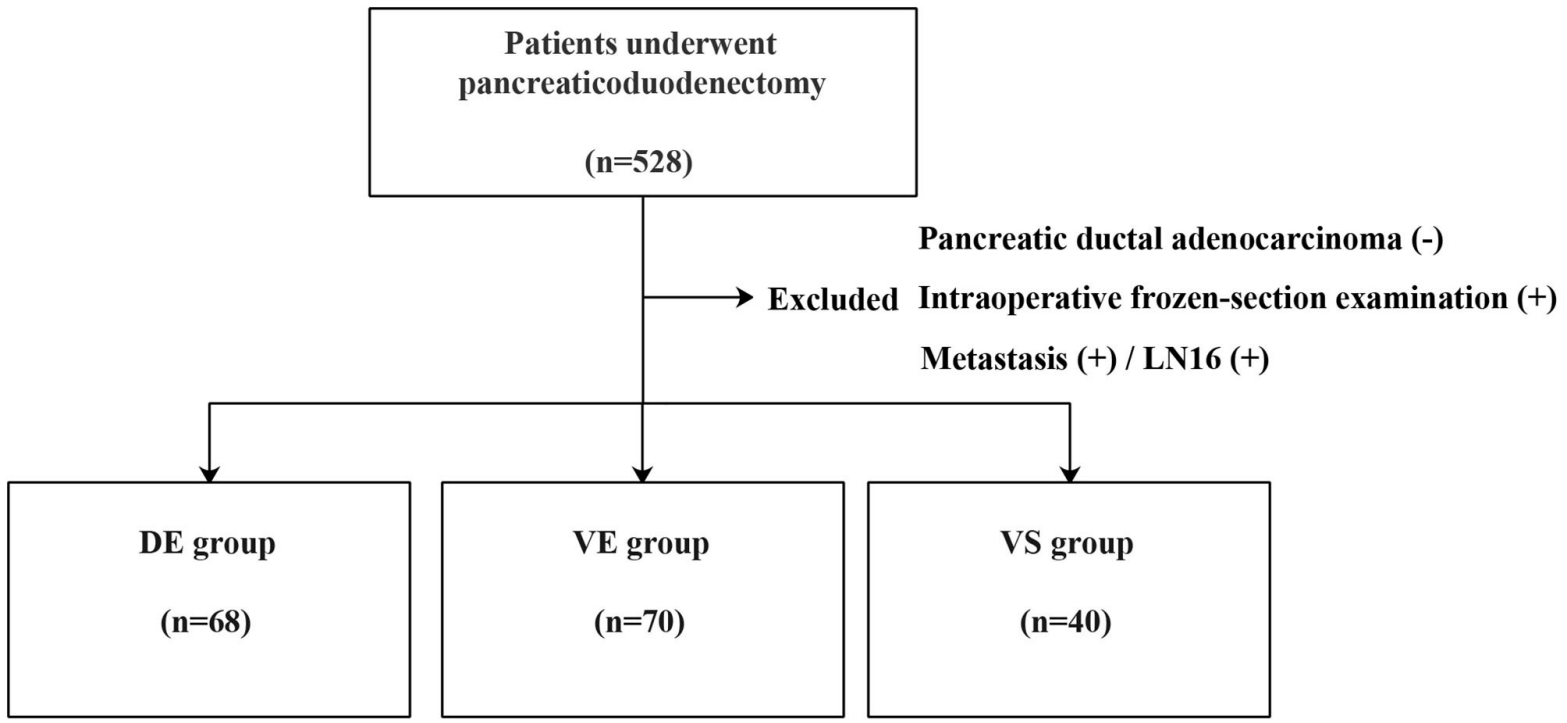
Flow chart of inclusion: *DE* patients with tumor located in the dorsal head of the pancreas performed with extended lymphadenectomy; *VE* patients with tumor located in the ventral head of the pancreas performed with extended lymphadenectomy; *VS* patients with tumor located in the ventral head of the pancreas performed with standard lymphadenectomy.

Finally, 178 patients were included in this study, including 70 patients with PDCA in the ventral primordium with extended lymphadenectomy (VE group), 40 patients with PDCA in the ventral primordium with standard lymphadenectomy (VS group), and 68 patients in the dorsal primordium with extended lymphadenectomy (DE group) (Figure 4).

### Assessment of Tumor Progression

Tumor stage was assessed using the eighth edition of the American Joint Committee on Cancer (AJCC) classification (8). The Japan Pancreas Society's General Rules for the Study of Pancreatic Cancer (6th edition, 2009) for LNs station was applied (9).

### Follow-Up Visit

Since discharge, follow-ups were performed with telephone interviews every 2 months, recording the time and location of recurrence and their survival. Disease-free survival (DFS) and overall survival (OS) time were calculated from the date of the operation to the date of tumor recurrence or death. The patients with tumor recurrence and death were considered as event data; patients with no tumor recurrence or death were classified as censored data. The patients lost to follow-up were classified based on the condition of the last follow-up.

### Statistical Analysis

All statistical analyses were performed using SPSS statistical software (version 22). Continuous variables were expressed as means with standard deviation or as medians with range or as rates (percentage). Continuous variables were compared using the Mann-Whitney *U* test. Categorical variables were compared using the chi-square test and the Fisher exact test in case of small expected frequencies. For the survival analysis, DFS and OS rates were analyzed by the Kaplan-Meier method with comparison of the log-rank test. For all tests, *P* < 0.05 were considered significant.

## Results

### Patient Characteristics

Of 178 patients included, 70 (39.3%) of patients were divided in the VE group, 68 (38.2%) in the DE group and 40 (22.5%) in the VS group. Patient demographic characteristics did not significantly differ among the three groups (Table 1), and neither did the preoperative tumor markers *including* carbohydrate antigen (CA) 19-9 and CA125. In preoperative imaging, common bile duct (CBD) dilation was identified significantly more frequently in the VE group (81.4 vs. 66.2%, *p* = 0.041) and the VS group (90.0 vs. 66.2%, *p* = 0.006) compared with the DE group (Table 1). Meanwhile there was no significant difference in the proportion of CBD dilation between the VE group and the VS group (*p* = 0.232). This was consistent with the previous study that carcinoma in the ventral head of the pancreas was more likely to lead to bile duct stenosis (6).

**Table 1:** Patient characteristics.

	**DE group**	**VE group**	**VS group**	
**Characteristics**	***n* = 68**	***n* = 70**	***n* = 40**	***P*-value**
**PATIENT DEMOGRAPHICS**				
Age, y	62 (44–84)	63 (35–85)	62 (42–87)	0.871
Sex, male	48 (70.6%)	44 (62.9%)	29 (72.5%)	0.489
**PREOPERATIVE FACTORS**				
Hb, g/L	126 (82–171)	127 (88–158)	125 (82–158)	0.983
PLT, 109/L	210 (71–435)	202 (73–383)	219 (98–390)	0.875
ALB, g/L	38 (24–50)	38 (26–51)	37 (29–54)	0.800
CA−199, U/mL	175.3 (0–17037)	156.9 (0–40200.0)	568.5 (0–8183.6)	0.696
CA−125, U/mL	15.3 (0.0–96.1)	16.3 (4.3–171.9)	27.8 (6.9–103.7)	0.385
TB, μmol/L	52.1 (6.6–407.8)	49.2 (6.2–416.4)	96.2 (7.0–292.4)	0.171
PBD	8 (11.8%)	5 (7.1%)	3 (7.5%)	0.594
Dilation of MPD	49 (72.1%)	44 (62.9%)	28 (70.0%)	0.487
Dilation of CBD^➀^	45 (66.2%)	57 (81.4%)	36 (90.0%)	0.010

➀ * Further intergroup χ test: DE group vs. VE group 66.2% vs. 81.4%, p = 0.041; DE group vs. VS group 66.2% vs. 90.0%, p = 0.006; VE group vs. VS group 81.4% vs. 90.0%, p = 0.232. Hb, Hemoglobin; PLT, Platelet; ALB, Albumin; TB, Total Bilirubin; PBD, preoperative biliary drainage*.

### Pathological Data and Tumor Stage

There was no significant difference in tumor diameter among the three groups (Table 2). Compared with the DE group, SMA in the VE group were more likely to be invaded (34.3 vs. 1.5%, *p* = 0.000), leading to a higher proportion of T4 tumor in the VE group than in the DE group (34.3 vs. 8.8% *p* = 0.000). There was no statistically significant difference in either SMA invasion or proportion of T4 tumor between the VE and VS groups (Supplementary Table 2). Patients in the DE group were associated with more portal vein (PV) invasion than the VE group (25.0 vs. 11.4%, *p* = 0.039). This may be related to the fact that the ventral pancreatic head tumor was more likely to be exposed to SMA in the anatomical position (6).

**Table 2:** Pathologic variables.

	**DE group**	**VE group**	**VS group**	
**Pathologic variables**	***n* = 68**	***n* = 70**	***n* = 40**	***P*–value**
Tumor size, cm	3.07 ± 1.16	3.19 ± 0.98	3.12 ± 0.96	0.700
SMA invasion	1 (1.5%)	24 (34.3%)	7 (17.5%)	0.000
CHA invasion	5 (7.4%)	3^➁^ (4.3%)	0 (0.0%)	0.092
SMV invasion	10 (14.7%)	19 (27.1%)	14 (35.0%)	0.045
PV invasion	17 (25.0%)	8 (11.4%)	2 (5.0%)	0.011
**T STAGE**				
T1	12 (17.6%)	8 (11.4%)	5 (12.5%)	0.547
T2	44 (64.7%)	29 (41.4%)	23 (57.5%)	0.020
T3	6 (8.8%)	9 (12.9%)	5 (12.5%)	0.795
T4	6 (8.8%)	24 (34.3%)	7 (17.5%)	0.001
**N STAGE**				
N0	39 (57.4%)	26 (37.1%)	20 (50.0%)	0.057
N1	23 (33.8%)	35 (50.0%)	15 (37.5%)	0.136
N2	6 (8.8%)	9 (12.9%)	5 (12.5%)	0.724
Total retrieved LNs	17.28 ± 5.17	22.50 ± 8.10	19.0 ± 5.91	0.000
No. positive LNs	1.09 ± 1.71	1.70 ± 1.81	1.35 ± 2.02	0.043
**AJCC STAGE (8TH EDITION)**				
IA	8 (11.8%)	6 (8.6%)	3 (7.5%)	0.720
IB	23 (33.8%)	12 (17.1%)	10 (25.0%)	0.079
IIA	3 (4.4%)	2 (2.9%)	3 (7.5%)	0.527
IIB	23 (33.8%)	22 (31.4%)	12 (30.0%)	0.910
III	11 (16.2%)	28 (40.0%)	12 (30.0%)	0.008
T4 (+) N2 (+)	0 (0.0%)	5 (17.9%)	0 (0.0%)	/
T4 (+) N2 (–)	5 (7.4%)	19 (27.1%)	7 (17.5%)	0.009
T4 (–) N2 (+)	6 (8.8%)	4 (5.7%)	5 (12.5%)	0.464

➁ *SMA was invaded by tumor at the same time for these three patients*.

There were significant differences in LNs detected, LNs, and the proportion of patients in stage III among the three groups (Table 2). More LNs were detected (22.50 ± 8.10 vs. 17.28 ± 5.17, *p* = 0.000) and were confirmed positive LN (1.70 ± 1.81 vs. 1.09 ± 1.71, *p* = 0.015) in the VE group than those in the DE group (Supplementary Table 1). More LNs were detected in the VE group than those in the VS group (22.50 ± 8.10 vs. 19.07 ± 5.91, *p* = 0.045) (Supplementary Table 2). And more patients in the VE group were divided in stage III than those in the DE group (40.0% vs. 16.2%, *p* = 0.002) (Supplementary Table 1). There were no statistically significant differences in the rest aspects (Supplementary Tables 1, 2).

### Location of Lymph Node Involvement

The peripancreatic LNs (LN13 and LN17) were the two main LNs involved in patients in these three groups. The proportion of LN14 metastases was significantly different among the three groups. Patients in the VE group were more likely to be involved with LN14 metastasis than patients in the DE group (22.9 vs. 5.9%, *p* = 0.005, in which LN14ab: 15.9 vs. 5.9%, *p* = 0.064, LN14cd: 10 vs. 0.0%, *p* = 0.022). The proportion of patients with LN14 metastasis was also significantly higher in the VE group than that in the VS group (22.9 vs. 5.0%, *p* = 0.015). There were no significant differences in LN metastasis in the rest of the locations. The positive rates of LN in each location of the three groups are shown in Table 3, Supplementary Table 3, and Table 4.

**Table 3:** Location of Lymph Node involvement in three groups.

	**DE group**	**VE group**	**VS group**	
	***n* = 68**	***n* = 70**	***n* = 40**	
**LN no**.	**Frequency of metastasis**			***P*-value**
5	2 (2.9%)	1 (1.4%)	0 (0.0%)	0.980
6	1 (1.5%)	1 (1.4%)	0 (0.0%)	1.000
8a	3 (4.4%)	2 (2.8%)	1 (2.5%)	0.832
8p	1 (1.5%)	1 (1.4%)	/	1.000^➂^
12	2 (2.9%)	3 (3.8%)	0 (0.0%)	0.423
12b + 12c	2 (2.9%)	1 (1.4%)	0 (0.0%)	0.980
12a + 12p	0 (0.0%)	1 (1.4%)	/	1.000^➂^
13	12 (17.6%)	20 (28.6%)	12 (30.0%)	0.225
14^➃^	4 (5.9%)	16 (22.9%)	2 (5.0%)	0.003
14ab	4 (5.9%)	11 (15.7%)	2 (5.0%)	0.003
14cd	0 (0.0%)	7 (10.0%)	/	0.022^➂^
17	13 (19.1%)	12 (17.1%)	9 (22.5%)	0.789

➂ *χ test between VE and DE group*.

➃ *DE group vs. VE group: LN14: 5.9 vs. 22.9%, p = 0.005, LN14ab: 5.9 vs. 15.9%, p = 0.064, LN14cd: 0.0 vs. 10.0%, p = 0.022). VE group vs. VS group, LN14: 22.9 vs. 5.0%, p = 0.015*.

**Table 4:** Perioperative risk and postoperative complications.

	**DE group**	**VE group**	**VS group**	***P*-value**
	***n* = 68**	***n* = 70**	***n* = 40**	
Postoperative fatality	1 (1.7%)	0 (0.0%)	2 (5.0%)	0.637
Reoperation (DSA)	3 (4.4%)	1 (1.4%)	1 (2.5%)	0.565
Pancreatic fistula	8 (11.8%)	6 (8.6%)	1 (2.5%)	0.246
Biliary fistula	1 (1.5%)	2 (2.9%)	3 (7.5%)	0.234
Gastric fistula	2 (2.9%)	0 (0.0%)	3 (7.5%)	0.072
Intra-abdominal abscess	9 (13.2%)	10 (14.3%)	2 (5.0%)	0.312
Delayed gastric emptying	2 (2.9%)	1 (1.4%)	0 (0.0%)	0.506
Intra-abdominal bleeding	3 (4.4%)	2 (2.9%)	2 (5.0%)	0.829
Ascites	0 (0.0%)	2 (2.9%)	1 (2.5%)	0.386
Operation time, min	298 (150–720)	303 (120–480)	301 (120–600)	0.445
Intraoperative bleeding, ml	436 (50–1,500)	342 (50–1,200)	427 (50–3,400)	0.053
Intraoperative transfusion	46 (67.6%)	41 (58.6%)	26 (65.0%)	0.528

Three groups of patients with LN14 metastasis were further analyzed in Supplementary Table 5 for details. In the 16 patients with LN14 metastasis in the VE group if only the LN14ab was dissected according to the standard LN dissection criteria, four (25.0%) patients with N1 stage would have been misclassified as N0. Besides, the preoperative characteristics and postoperative pathology of the VE group did not differ from those of the VS group except for the positive rate of LN14, suggesting that the LN dissection of the right side of SMA (LN14ab) may not be sufficient for patients with PDAC located in the ventral head of the pancreas.

### Perioperative Risk

The postoperative mortality, reoperation rate, complications, duration of operation, intraoperative bleeding, and intraoperative transfusion of these three groups are shown in Table 4. In this study, three of the 178 patients experienced postoperative nosocomial death. Among them, one patient in the DE group died of pancreatic fistula in the ward on the 10th day after the operation. One patient in the VS group died of abdominal hemorrhage on the sixth day after the operation, and another patient in the VS group died due to SMA embolization on the seventh day after surgery. One patient in the VE group and three patients in the DE group received reoperation for postoperative hemorrhage, and one patient in the VS group who was suspected to be complicated with postoperative hemorrhage underwent laparotomy. There was no significant difference in the incidence of postoperative complications, including pancreatic fistula, biliary fistula, and delayed gastric emptying. Therefore, we propose that extended lymphadenectomy may not increase the perioperative risk.

### Tumor Recurrence

Liver was the main site of tumor recurrence in the three groups. Although the proportion of patients with LN14 metastases in the VE group was higher than that in the other two groups, there was no significant difference in the rate of recurrence around SMA, which may be attributed to the thorough dissection of the surrounding SMA during the operation. The rest of the tumor recurrences are shown in Table 5, and there was no significant difference.

**Table 5:** Recurrence pattern.

	**DE group**	**VE group**	**VS group**	***P*-value**
	***n* = 68**	***n* = 70**	***n* = 40**	
**RECURRENCE**				
Residual pancreas	3 (4.4%)	2 (2.9%)	2 (5.0%)	0.829
SMA	2 (2.9%)	8 (11.4%)	2 (5.0%)	0.122
Liver	22 (32.3%)	33 (47.1%)	15 (37.5%)	0.198
Lung	3 (4.4%)	2 (2.9%)	1 (2.5%)	0.829
Bone	1 (1.4%)	1 (1.4%)	2 (5.0%)	0.411
Peritoneal seeding	8 (11.8%)	6 (8.6%)	3 (7.5%)	0.720
Retroperitoneal lymph node	4 (5.9%)	5 (7.1%)	2 (5.0%)	0.897
Others	0 (0.0%)	0 (0.0%)	1 (2.5%)	/

*SMA superior mesenteric artery Others distal lymph node metastasis*.

### Survival Analysis

The rate of patients lost to follow-up was 2.7% with two patients in the VE group and one patient in the VS group. The minimal follow-up time was 15.4 months without tumor recurrence or death as censored data. The median follow-up time was 28.6 months.

In general, the range of lymph node dissection did not make statistical differences on the prognosis of 110 patients with tumor in the ventral head of the pancreas. The median survival time (MST) in the VS group was 17.0 months, the 1-year survival rate (1-YSR) was 67.5%, and the median disease-free survival time (MDFST) was 10.8 months. The MST in the VE group was 16.9 months, the 1-YSR was 67.1%, and the MDFST was 10.2 months. Except for the extent of LN dissection, the univariate survival analysis results show that preoperative albumin level, total bilirubin level, tumor marker, dilation of main pancreatic duct or common bile duct, preoperative biliary drainage, intraoperative vein reconstruction, N stage, and LN14 (±) did not make a difference on OS and DFS time (Tables 6, 7) although the MDFST of patients with a T4 stage tumor was shorter than those with not-T4 stage (8.3 months vs. 12.7 months, *p* = 0.020). Further analysis showed that T4 stage was an independent prognostic factor of DFS [hazard ratio (HR) = 0.556, 95% confidence interval (CI): 0.337–0.918, *p* = 0.022].

**Table 6:** Prognostic factors in Univariate Analysis (OS).

	**Univariate analysis**			
	**Patients (*n*)**	**mOS (month)**	**1-YSR (%)**	***P*-value**
OP extent, standard/extended	40/70	17.0/16.9	67.5%/67.1%	0.598
ALB, <35/≥35 g/L	26/84	18.0/16.6	80.8%/63.1%	0.266
TB, <24/≥24 μmol/L	40/70	21.7/15.2	70.0%/65.7%	0.064
Preoperative CA-199, <37/≥37 (U/ml)	24/86	21.716.3	70.8%/66.3%	0.319
Preoperative CA-125, <35/≥35 (U/ml)	87/23	16.8/21.1	67.8%/65.2%	0.940
Dilation of MPD, no/yes	38/72	20.8/16.6	68.4%/66.7%	0.057
Dilation of CBD, no/yes	17/93	25.4/16.5	82.4%/64.5%	0.162
PBD, no/yes	102/8	16.8/22.0	65.7%/87.5%	0.330
Portal vein /SMV resection, No/Yes	96/14	17.1/14.9	63.5%/92.9%	0.510
T stage T4, positive/negative	31/79	17.8/16.2	67.6%/67.1%	0.335
N stage, N0/N1/N2	44/52/14	18.0/14.8/ 23.2	70.5%/63.5%/ 71.4%	0.283
LN14-/LN14+	92/18	16.8/16.9	69.6%/55.6%	0.436

*mOS median Overall Survival, mOS median overall survival, 1-YSR 1-year survival rate, OP operation, TB Total Bilirubin, PBD preoperative biliary drainage*.

**Table 7:** Prognostic factors in Univariate and Multivariate Analysis (DFS).

	**Univariate analysis**			**Multivariate analysis**		
	**Patients** **(*n*)**	**mDFS** **(m)**	***P*-value**	**HR**	**95%** **CI**	***P*-value**
OP extent, standard/extended	40/70	10.8/10.2	0.108			
ALB^➄^, <35/≥35 g/L	26/84	/	/			
TB, <24/≥24 μmol/L	40/70	9.4/11.1	0.275			
Preoperative CA-199, <37/≥37 (U/ml)	24/86	14.4/10.0	0.189			
Preoperative CA-125, <35/≥35 (U/ml)	87/23	10.6/10.4	0.639			
Dilation of MPD, no/yes	38/72	12.8/10.5	0.774			
Dilation of CBD, no/yes	17/93	9.1/11.3	0.092			
PBD, no/yes	102/8	10.7/12.7	0.784			
Portal vein /SMV resection, No/Yes	96/14	11.7/8.7	0.109			
T stage T4, positive/negative	31/79	8.3/12.7	0.020	0.556	0.337–0.918	0.022
N stage, N0/N1/N2+	44/52/14	12.8/9.4/9.7	0.285			
LN14-/LN14+	92/18	11.0/9.1	0.191			

➄ * More than 50% of data is censored. mDFS median disease-free survival time, HR Hazard Ratio, CI Confidence Interval*.

### Subgroup Analysis

**Figure 5:**
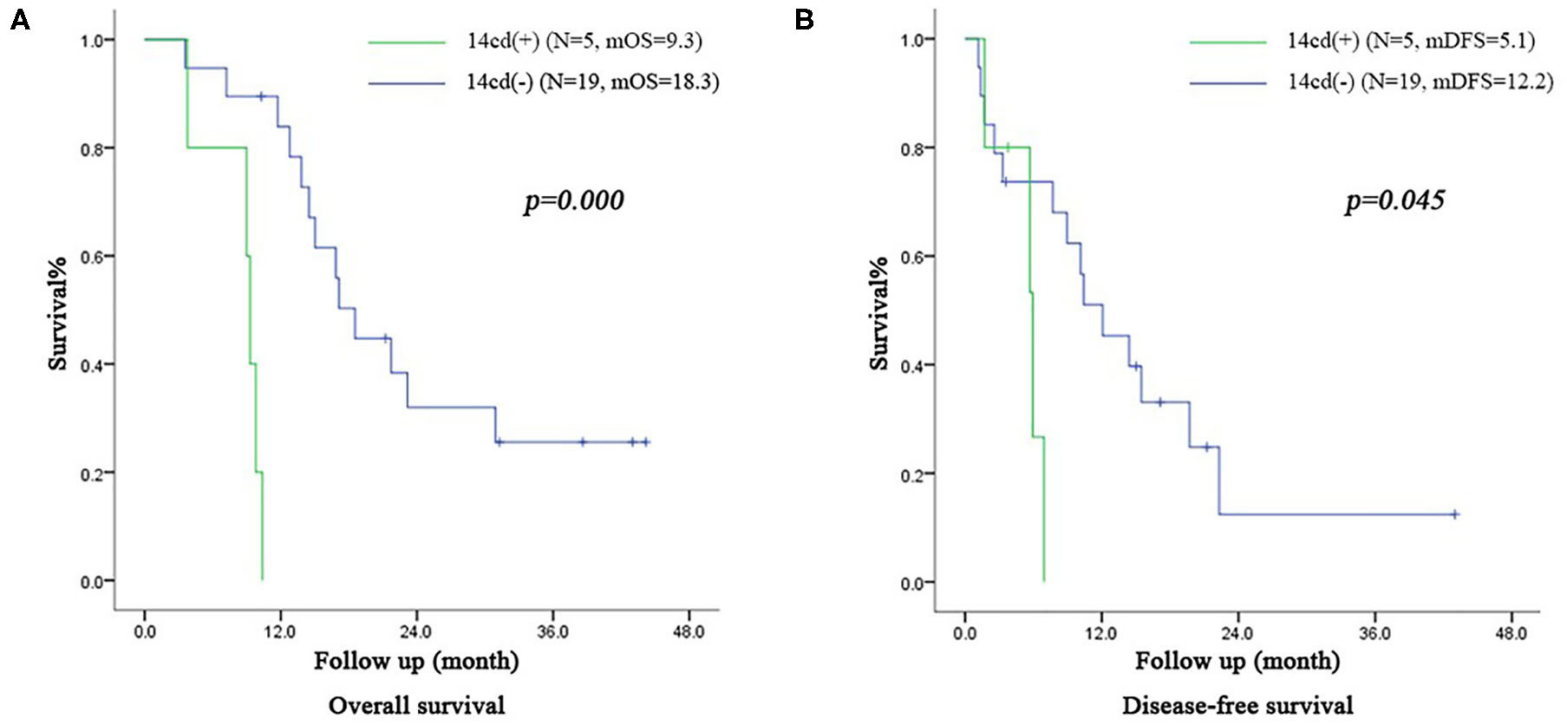
**(A)** Survival curve of subgroup (patients with IIb stage including LN(+) by direct tumor extension in VE group) according to the LN14cd (±), *mOS* median overall survival **(B)** Disease-free survival curve of subgroup (patients with IIb stage including LN(+) by direct tumor extension in VE group) according to the LN14cd (±), *mDFS* median disease-free survival.

In the subgroup of patients in the VE group with IIb stage, the OS time of patients with LN14cd (+) and the DFS time were both shorter than those with LN14cd (–) (OS: 9.3 months vs. 18.3 months, *p* = 0.000, DFS: 5.1 months vs. 12.2 months, *p* = 0.045; Figure 5).

## Discussion

Standard lymphadenectomy has been proven to prolong the 5-years survival rate of patients with PDCA in the head of the pancreas (10, 11), and it is the only criteria widely recognized by all at present (12). The necessity and the extent of extended lymphadenectomy remain a fierce debate. A few prospective clinical studies found that extended lymphadenectomy did not contribute to survival (13–16), thus optimization of the lymphadenectomy to obtain an accurate LN stage of pancreatic head cancer and ensure the safety of the operation is a major challenge. The International Study Group on Pancreatic Surgery (ISGPS) proposed a standard lymphadenectomy based on the positive rate of each LN station involved and the related prognostic significance (12). The dissection of regional LNs around the SMA remains controversial. As reported previously, LN14ab was associated with early recurrence (17), and the skeletonization of the right side of SMA contributed to isolate the uncinate during operation although skeletonization of the left side of SMA may significantly increase the surgical risk and the incidence of severe complications (18, 19). Therefore, dissection of LN14cd is not recommended in general.

This research suggests that the LN on both sides of SMA (LN14ab and LN14cd) should be thoroughly dissected for patients with resectable PDAC located in the ventral head of the pancreas, and for those with PDAC located in the dorsal head of the pancreas, only LN14ab should be dissected as the standard procedure.

First, the LN reflux of the head of the pancreas may circulate in different ways. Kitagawa et al. (6) proposed that the lymphatic pathways of the pancreatic head of different embryonal origin were not identical, and the tumors in the ventral head of the pancreas were more likely to metastasize to the LN14 although Okamura et al. (19) found that the positive rate of LN14 did not differ according to the embryonic segment of the head of the pancreas. The conclusion had certain limitations because the study excluded patients with tumor size >4 cm, and its study subjects were mainly patients with stage IIA and IIB. Besides the positive rate of LN14 was recorded as a whole instead of separating into LN14ab and LN14cd.

In this study, the pathological stage of 178 patients included varied from stage I to III according to current clinical guidelines. The results show that the number of positive LNs detected in the VE group was significantly higher than that in the DE group, and the difference was mainly contributed by the higher positive rate of LN14ab and LN14cd. With similar preoperative characteristics, the positive rate of LN14 was higher in the VE group compared with the VS group, and the rest were not significantly different. Furthermore, four of 16 patients in the VE group with LN14 metastasis would have been misclassified as N0 without LN dissection, including LN14cd. Meanwhile, patients with isolated LN14cd metastasis were found in previous studies (20). As a conclusion, this study suggests that there would be a high risk of both LN14ab and LN14cd metastasis in patients with PDAC located in the ventral head of the pancreas. Thus, positive LN14cd may be missed under standard lymphadenectomy with dissection of LN14ab, leading to the inaccurate tumor stage and the overestimation of prognosis.

In addition, corresponding to a recent study by Kenjiro et al. (21) proposing LN14cd metastasis as an independent risk factor for prognosis, the survival analysis of this study also suggests that LN metastasis in LN14cd would be an adverse prognostic factor for IIb patients with PDAC located in the ventral head of the pancreas. Because LN14cd was out of the range for standard lymphadenectomy and not commonly dissected during PD, few studies were concerned with LN14cd metastasis in pancreatic head cancer. The survival benefit of LN14cd dissection or prognostic value of LN14cd metastasis were not so clear as para-aortic lymph node (LN16), which was defined as the third station LNs according to the definition of the Japan Pancreas Society, equivalent to distant metastases and previous randomized controlled trials (RCT) pointed out that patients could not benefit from dissection of LN16 (22, 23). Other studies suggest that patients with LN16 metastasis confirmed during surgical exploration undergo neoadjuvant treatment instead of continuing exploration (24). To better understand the prognostic effect of LN14cd, further studies, including larger number of patients, especially those with ventral pancreatic head cancer with LN14cd dissection, would be needed.

Although patients with borderline tumor (T4 stage) were shown to benefit from neoadjuvant therapy with prolonged survival time, these patients may develop complications that may contradict with surgery, and tumors unresponsive to neoadjuvant therapy may become unresectable (25–28). Thus, the optimal treatment strategies for borderline tumor of the head of the pancreas are still under discussion. We propose that the necessity of LN14cd dissection for borderline PDAC needs to be further validated.

However, this retrospective study also has some limitations. Fewer patients were included in the VS group in this study than those in the VE group and the positive rate of LN14cd was low, which led to selection bias. Second, a more precise criteria to divide tumor by imaging according to embryonic origin would be explored. A larger number of patients should be included to further elucidate the prognostic effect of LN14cd.

## Data Availability Statement

The raw data supporting the conclusions of this article will be made available by the authors, without undue reservation.

## Ethics Statement

The studies involving human participants were reviewed and approved by Ruijin Hospital Ethics Committee. The patients/participants provided their written informed consent to participate in this study.

## Author Contributions

LQ, JuX, and ZX: study conception, drafting, and writing of the manuscript, tables. LQ, ZX, and WW: design and drawing figures. LQ, JuX, XD, HC, WC, and JiX: acquisition of data. LQ, JuX, ZX, CP, HL, and WW: analysis of data. JiX, WW, and BS: critical revision. All authors contributed to the article and approved the submitted version.

## Conflict of Interest

The authors declare that the research was conducted in the absence of any commercial or financial relationships that could be construed as a potential conflict of interest.
