# Supplementary material for: The Necessity of Dissection of No. 14 Lymph Nodes to Patients With Pancreatic Ductal Adenocarcinoma Based on the Embryonic Development of the Head of the Pancreas
Source: Front Oncol. 2020 Aug 11;10:1343. doi: 10.3389/fonc.2020.01343 (PMC7433687; doi:10.3389/fonc.2020.01343)
Supplement: Supplementary file 1 [file Data_Sheet_1.docx]

Supplementary Table1. Pathologic Variables between DE and VE group

|  | DE group | VE group |  |
| --- | --- | --- | --- |
| Pathologic variables | *n*= 68 | *n*= 70 | *P*-value |
| Tumor size, cm | 3.07±1.16 | 3.19±0.98 | 0.399 |
| SMA invasion | 1(1.5%) | 24(34.3%) | 0.000 |
| CHA invasion | 5(7.4%) | 3^①^(4.3%) | 0.684 |
| SMV invasion | 10(14.7%) | 19(27.1%) | 0.073 |
| PV invasion | 17(25.0%) | 8(11.4%) | 0.039 |
| T stage |  |  |  |
| T1 | 12(17.6%) | 8(11.4%) | 0.300 |
| T2 | 44(64.7%) | 29(41.4%) | 0.006 |
| T3 | 6(8.8%) | 9(12.9%) | 0.447 |
| T4 | 6(8.8%) | 24(34.3%) | 0.000 |
| N stage |  |  |  |
| N0 | 39(57.4%) | 26(37.1%) | 0.017 |
| N1 | 23(33.8%) | 35(50.0%) | 0.054 |
| N2 | 6(8.8%) | 9(12.9%) | 0.447 |
| Total retrieved LNs | 17.28±5.17 | 22.50±8.10 | 0.000 |
| No.positive LNs | 1.09±1.71 | 1.70±1.81 | 0.015 |
| AJCC stage(8th edition) |  |  |  |
| ⅠA | 8(11.8%) | 6(8.6%) | 0.534 |
| ⅠB | 23(33.8%) | 12(17.1%) | 0.024 |
| ⅡA | 3(4.4%) | 2(2.9%) | 0.974 |
| ⅡB | 23(33.8%) | 22(31.4%) | 0.764 |
| III | 11(16.2%) | 28(40.0%) | 0.002 |
| T4(+) N2(+) | 0(0.0%) | 5(17.9%) | / |
| T4(+) N2(-) | 5(7.4%) | 19(27.1%) | 0.002 |
| T4(-) N2(+) | 6(8.8%) | 4(5.7%) | 0.707 |

① SMA was invaded by tumor at the same time for these three patients

Supplementary Table2. Pathologic Variables between VE and VS group

|  | VE group | VS group |  |
| --- | --- | --- | --- |
| Pathologic variables | *n*= 70 | *n*= 40 | *P*-value |
| Tumor size, cm | 3.19±0.98 | 3.12±0.96 | 0.721 |
| SMA invasion | 24(34.3%) | 7(17.5%) | 0.060 |
| CHA invasion | 3(4.3%) | 0(0.0%) | 0.459 |
| SMV invasion | 19(27.1%) | 14(35.0%) | 0.387 |
| PV invasion | 8(11.4%) | 2(5.0%) | 0.433 |
| T stage |  |  |  |
| T1 | 8(11.4%) | 5(12.5%) | 1.000 |
| T2 | 29(41.4%) | 23(57.5%) | 0.104 |
| T3 | 9(12.9%) | 5(12.5%) | 0.957 |
| T4 | 24(34.3%) | 7(17.5%) | 0.060 |
| N stage |  |  |  |
| N0 | 26(37.1%) | 20(50.0%) | 0.188 |
| N1 | 35(50.0%) | 15(37.5%) | 0.205 |
| N2 | 9(12.9%) | 2(5.0%) | 0.322 |
| Total retrieved LNs | 22.50±8.10 | 19.07±5.91 | 0.045 |
| No.positive LNs | 1.70±1.81 | 1.35±2.02 | 0.138 |
| AJCC stage(8th edition) |  |  |  |
| ⅠA | 6(8.6%) | 3(7.5%) | 1.000 |
| ⅠB | 12(17.1%) | 10(25.0%) | 0.322 |
| ⅡA | 2(2.9%) | 3(7.5%) | 0.516 |
| ⅡB | 22(31.4%) | 12(30.0%) | 0.876 |
| III | 28(40.0%) | 12(30.0%) | 0.294 |
| T4(+) N2(+) | 5(17.9%) | 0(0.0%) | / |
| T4(+) N2(-) | 19(27.1%) | 7(17.5%) | 0.252 |
| T4(-) N2(+) | 4(5.7%) | 5(12.5%) | 0.375 |

Supplementary Table3. Location of Lymph Node involvement between DE and VE groups

|  | DE group | VE group |  |
| --- | --- | --- | --- |
|  | *n*=68 | *n*=70 |  |
| LN no. | Frequency of Metastasis | | *P*-value |
| 5 | 2(2.9%) | 1(1.4%) | 0.980 |
| 6 | 1(1.5%) | 1(1.4%) | 1.000 |
| 8a | 3(4.4%) | 2(2.8%) | 0.974 |
| 8p | 1(1.5%) | 1(1.4%) | 1.000 |
| 12 | 2(2.9%) | 3(3.8%) | 1.000 |
| 12b+12c | 2(2.9%) | 1(1.4%) | 0.980 |
| 12a+12p | 0(0.0%) | 1(1.4%) | 1.000 |
| 13 | 12(17.6%) | 20(28.6%) | 0.128 |
| 14 | 4(5.9%) | 16(22.9%) | 0.005 |
| 14ab | 4(5.9%) | 11(15.7%) | 0.064 |
| 14cd | 0(0.0%) | 7(10.0%) | 0.022 |
| 17 | 13(19.1%) | 12(17.1%) | 0.763 |

Supplementary Table4. Location of Lymph Node involvement between VE and VS groups

|  | VE group | VS group |  |
| --- | --- | --- | --- |
|  | *n*=70 | *n*=40 |  |
| LN no. | Frequency of Metastasis | | *P*-value |
| 5 | 1(1.4%) | 0(0.0%) | 1.000 |
| 6 | 1(1.4%) | 0(0.0%) | 1.000 |
| 8a | 2(2.8%) | 1(2.5%) | 1.000 |
| 8p | 1(1.4%) | / | / |
| 12 | 3(3.8%) | 0(0.0%) | 0.472 |
| 12b+12c | 1(1.4%) | 0(0.0%) | 1.000 |
| 12a+12p | 1(1.4%) | / | / |
| 13 | 20(28.6%) | 12(30.0%) | 0.874 |
| 14 | 16(22.9%) | 2(5.0%) | 0.015 |
| 14ab | 11(15.7%) | 2(5.0%) | 0.171 |
| 14cd | 7(10.0%) | / | / |
| 17 | 12(17.1%) | 9(22.5%) | 0.308 |

Supplementary Table 5. Details of patients with LN14 metastasis

|  | Patient | LN14ab(+)/LN14ab retrieved | LN14cd(+)/LN  14cd retrieved | N stage | N’ stage |
| --- | --- | --- | --- | --- | --- |
| VE group | (1) | 0/0 | 2/4 | N1 | N0 |
|  | (2) | 2/10 | 0/0 | N1 | N1 |
|  | (3) | 1/3 | 0/0 | N2 | N2 |
|  | (4) | 1/1 | 0/1 | N1 | N1 |
|  | (5) | 2/3 | 0/0 | N1 | N1 |
|  | (6) | 1/3 | 0/4 | N1 | N1 |
|  | (7) | 1/4 | 2/6 | N1 | N1 |
|  | (8) | 1/5 | 0/0 | N1 | N1 |
|  | (9) | 1/1 | 0/0 | N1 | N1 |
|  | (10) | 1/3 | 0/0 | N2 | N2 |
|  | (11) | 1/5 | 1/5 | N1 | N1 |
|  | (12) | 0/5 | 2/2 | N1 | N0 |
|  | (13) | 1/2 | 0/0 | N1 | N1 |
|  | (14 | 0/2 | 1/1 | N1 | N0 |
|  | (15) | 0/0 | 1/1 | N1 | N1 |
|  | (16) | 0/2 | 1/5 | N1 | N0 |
| VE group | (1) | 2/7 | 0/3 | N1 | / |
|  | (2) | 2/2 | 0/0 | N1 | / |
|  | (3) | 2/2 | 0/0 | N1 | / |
|  | (4) | 2/2 | 0/0 | N1 | / |
| VS group | (1) | 1/2 | / | N1 | / |
|  | (2) | 1/3 | / | N1 | / |

*N’ stage* N stage without dissection of LN14cd *SMA* Superior mesenteric artery
